# miRNA-106a directly targeting RARB associates with the expression of Na^+^/I^−^ symporter in thyroid cancer by regulating MAPK signaling pathway

**DOI:** 10.1186/s13046-016-0377-0

**Published:** 2016-06-24

**Authors:** Chen-Tian Shen, Zhong-Ling Qiu, Hong-Jun Song, Wei-Jun Wei, Quan-Yong Luo

**Affiliations:** Department of Nuclear Medicine, Shanghai Jiao Tong University Affiliated Sixth People’s Hospital, 600 Yishan Road, Shanghai, 200233 People’s Republic of China

**Keywords:** miR-106a, RARB, Sodium-iodide symporter, Thyroid cancer, MAPK signaling pathway

## Abstract

**Background:**

Serum miRNAs profiles between papillary thyroid carcinoma (PTC) patients with non-^131^I and ^131^I-avid lung metastases are differentially expressed. These miRNAs have to be further validated and the role of these miRNAs in the molecular function level of thyroid cancer cell lines has not been investigated.

**Methods:**

Expression levels of six identified miRNAs were assessed via quantitative real-time PCR (qRT-PCR) in the serum of eligible patients. Dual-luciferase reporter assay was used to determine the potential target of miR-106a. Cell viability and apoptosis were evaluated by MTT assay and flow cytometry analysis, respectively. The change of gene expression was detected by qRT-PCR and western blotting analysis. In vitro iodine uptake assay was conducted by a γ-counter.

**Results:**

Compared to PTC patients with ^131^I-avid lung metastases, miR-106a was up-regulated in the serum of patients with non-^131^I-avid lung metastases. The results of dual-luciferase reporter assay demonstrated that miR-106a directly targeted retinoic acid receptor beta (RARB) 3′-UTR. miR-106a-RARB promoted viability of thyroid cancer cells by regulating MEKK2-ERK1/2 and MEKK2-ERK5 pathway. miR-106a-RARB inhibited apoptosis of thyroid cancer cells by regulating ASK1-p38 pathway. Moreover, miR-106a-RARB could regulate the expression of sodium iodide symporter, TSH receptor and alter the iodine uptake function of thyroid cancer cells.

**Conclusions:**

miRNA-106a, directly targeting RARB, associates with the viability, apoptosis, differentiation and the iodine uptake function of thyroid cancer cell lines by regulating MAPK signaling pathway in vitro. These findings in the present study may provide new strategies for the diagnosis and treatment in radioiodine-refractory differentiated thyroid carcinoma.

## Background

Differentiated thyroid cancer (DTC) is increasing all over the world [1], as an indolent tumor, DTC patients have excellent prognosis following conventional treatments based on adequate surgical management, radioactive iodine (RAI) ablation and thyroid-stimulating hormone (TSH) suppression [2, 3]. Radioiodine is considered to be the initial systemic and efficient treatment for metastatic DTC patients. Unfortunately, approximately 30 % of patients with advanced, metastatic DTC have radioiodine-refractory disease [4]. Losing the ability to concentrate radioiodine in metastatic sites from DTC most likely owns to less differentiated types transformation (dedifferentiation) [5]. This problem creates a major obstacle in radioiodine treatment for those patients while the mechanisms underlying the dedifferentiation transformation of DTC are still not well understood.

It is well accepted that constitutive activation of mitogen-activated protein kinase (MAPK) signaling pathway plays a significant role in the tumorigenesis of thyroid carcinoma and it also could promote the dedifferentiation of thyroid-cancer cells. Regarding to this fact, disease-specific molecular targets of therapy is studied much popularly [6]. MAP3K2 (MEKK2) is a serine/threonine kinase that belongs to the MEKK/STE11 family of MAP kinase kinase kinases, which can activate JNK1/2 [7], p38 [8], ERK5 [9] and ERK1/2 [10] pathways. However, its role in thyroid cancer has not been clearly studied by now.

Recently, the involvement of miRNAs in proliferation, differentiation and apoptosis has been defined and several reports have displayed the changes in miRNA profiles in differentiated thyroid cancers as compared to normal thyroid tissues [11–15]. But the role of miRNAs in the differentiation/dedifferentiation of DTC, especially in the expression of sodium iodide symporter (NIS) and NIS-mediated iodine uptake, is not clearly understood. Lakshmanan et al. found that miR-339-5p directly bound to hNIS-3′UTR and miR-339-5p overexpression decreased NIS-mediated radioiodine uptake in HEK293 cells expressing exogenous hNIS [16].

One of our previous studies analyzed the differentially expressed serum miRNAs profiles between papillary thyroid carcinoma (PTC) patients with non-^131^I and ^131^I-avid lung metastases [17]. But the function of these miRNAs in thyroid cancer has not been reported. In the current study, the role of miRNA-106a in the viability, apoptosis, migration, invasion and differentiation (focused on the expression of NIS and TSH receptor and the ability of iodine uptake) of thyroid cancer cell lines was investigated.

## Methods

### Serum samples and cell culture

The serum samples of PTC patients with ^131^I-avid and non-^131^I-avid lung metastases were collected from September 2010 to July 2014 at our department. The inclusion/exclusion criteria and the method to process the samples have been described before [17]. Total RNA from serum sample was extracted and purified using the miRNeasy Mini Kit (Qiagen, Valencia, CA) according to the manufacturer’s instructions. The study was approved by the Institutional Ethics Review Board of our hospital and informed consent was obtained from each patient.

Human PTC cell line CGTH-W3 (Institute of Biochemistry and Cell Biology, SIBS, CAS, Shanghai, China) and anaplastic thyroid carcinoma (ATC) cell line 8505C (Institute of Biochemistry and Cell Biology, SIBS, CAS, Shanghai, China) were cultured in RPMI-1640 (Gibco, Cat. # 11875–093) supplemented with 10 % fetal bovine serum (Gibco, Cat. # 16000–044). HEK 293 T (Institute of Biochemistry and Cell Biology, SIBS, CAS, Shanghai, China) was maintained in Deulbecco’s Modified Eagle medium (Gibco, Cat. # 10565–018) supplemented with 10 % fetal bovine serum (Gibco, Cat. # 16000–044). All the cells were incubated at 37 °C in a humidified chamber supplemented with 5 % CO_2_. U0126 and SB203580 were obtained from Selleck Chemicals (Houston, TX, Cat. # S1102 and S1076). All-trans retinoic acid (RA) was obtained from Sigma-Aldrich (Cat. # R2625).

### Different managements of cells

#### 8505C-miR106a(−) and 8505C-scrambled control

miArrest™ miRNA inhibitor (GeneCopoeia, Cat. # HmiR-AN0026-AM03), scrambled control (GeneCopoeia, Cat. # CmiR-AN0001-AM03) and Lenti-Pac™ HIV Expression Packaging Kit (GeneCopoeia, Cat. # HPK-LvTR-20) were used to construct Lenti-Pac-miRNA-106a inhibitor and Lenti-Pac- scrambled control. After transfected to packaging cells (HEK 293T), the medium was collected and centrifuged at 4,000 × g for 10 min at room temperature to pellet cell debris, and then filtered through a 0.45 μm filter. Target cells (8505C) were transfected with Lenti-Pac-miRNA-106a inhibitor [8505C-miR106a(−)] or Lenti-Pac-scrambled control (8505C-scrambled control).

#### 8505C-miR106a(−) + RARB(−)

8505C-miR106a(−) cells were transfected with Human retinoic acid receptor beta (RARB) Silencer® Select siRNA (Ambion, target sequence: TCAGACGGCCTTACCCTAAAT, Cat. # 4392420) using Lipofectamine 2000 (Invitrogen, USA, Cat. # 11668019) following the manufacturer’s instructions.

#### 8505C-miR106a(−) + ASK1(−)

Selective inhibitor of ASK1, (2,7-dihydro-2,7-dioxo-3H-naphtho [1,2,3-de] quinoline-1-carboxylic acid ethyl ester; NQDI-1) (Sigma, Cat. # SML0185), was used to inhibit ASK1 by pretreating the 8505C-miR106a (−) cells.

#### 8505C-miR106a(−) + MAP3K2(+)

8505C-miR106a(−) cells were transiently transfected with MAP3K2 (NM_006609) Human cDNA ORF Clone (OriGene, Cat. # RC223988) using 100 μl Opti-MEM I (Gibco, Cat. # 51985091) and 3 μL Turbofectin 8.0 (OriGene, Cat. # TF81001).

#### CGTH-W3-miR106a(+) and CGTH-W3-*control vector*

The amplified human miR-106a gene was digested with BamHI and EcoRI restriction enzymes and was cloned into the lentiviral vector pCDH-CMV-MCS-EF1-copGFP (System Biosciences, Cat. # CD511B-1). The pCDH-miR106a lentiviral vector was transformed into *E. coli* DH5α, then pCDH-miR106a was purified using a plasmid kit (Qiagen, Cat. # 12143) according to the manufacturer’s instructions. Then pCDH-miR106a was packaged into HEK 293 T cells with the pPACK packaging mix using a lentivector expression system (System Biosciences) according to the manufacturer’s instructions. After transfection to packaging cells for 48 h, the medium was collected and centrifuged at 4,000 × g for 10 min at room temperature to pellet cell debris, and then filtered through a 0.45 μm filter. Target cells (CGTH-W3) were transfected with Lenti-pCDH-miR106a [CGTH-W3-miR106a(+)] or Lenti-pCDH (CGTH-W3-control vector).

#### CGTH-W3-RARB(−)

CGTH-W3 cells were transfected with Human RARB Silencer® Select siRNA (Ambion, target sequence: TCAGACGGCCTTACCCTAAAT) using Lipofectamine 2000 (Invitrogen, Cat. # 11668019) following the manufacturer’s instructions.

### Dual-luciferase reporter assay

A fragment of 3′UTR of RARB (NM_000965) containing the putative miR-106a binding sites was amplified by PCR using the following primers:

- wt- RARB (forward): 5′- GGGTACCCCTACTTCAAACATTCCCCAG-3′;
- wt- RARB (reverse): 5′-CCCTCGAGGGTGAGAACTAAGAAACTGACA-3′;
- 3′UTR of RARB with a mutant seed sequence of miR-106a was synthesized using the following:
- mut-RARB (forward): 5′-GGGTACCCTTCAAACATTCCCCAGTACCTTCAGT-3′;
- mut-RARB(reverse): 5′-CCCTCGAGGGTTTTAATTTAAGCGCACATTAACAAT-3′;

Then pGL3-RARB 3′UTR–wt, pGL3-RARB 3′UTR-mut vectors were constructed. For the reporter assay, HEK 293T cells were plated into 24-well plates and co-transfected with the above constructs and miR-106a mimics/miR-negative controls using the Lipofectamine 2000 reagent (Invitrogen, Cat. # 11668019). After 48 h, the cells were harvested and assayed using the dual-Luciferase Reporter Assay system (Promega, Cat. # E1910) according to the manufacturer’s instructions.

### MTT: cell viability assays

The cell viability were evaluated using 3-(4, 5-dimethylthiazol-2-yl) 22, 5-diphenyltetrazolium bromide (MTT) assay. Cells were seeded in sextuplicate in 96-well microtiter plates at a density of 1 × 10^4^ cells/well in 100 μL medium. The plates were incubated in a 37 °C humidified incubator for adherence overnight. Then after 0, 24, 48, 72 and 96 h culture, 20 μL of 5 g/L MTT (Amresco, Cat. # 0793-500MG) was added, respectively. The medium was removed after 4 h, and the reaction was then stopped by the addition of DMSO and measured at A570 in a Microplate spectrophotometer (Spectra Max Plus, Molecular Devices, Sunnyvale, CA). The results were expressed as percentage, based on the ratio of the absorbance between the treated cells and the controls (100 %). Experiments were repeated three times.

### Apoptosis flow cytometry analysis

ApoAlert Annexin V-FITC kit (Clontech, Cat. # 630109) was used to assess the cell apoptosis. Parental CGTH-W3 and 8505C cells and transfected sublines were seeded in 6-well plates at 1 × 10^5^ per well. Cells were harvested 72 h later and stained with Annexin V-FITC and propidium iodide according to the manufacturer’s protocol. Cell samples were analyzed on a FACScan Analyzer and apoptotic fractions were determined. Experiments were repeated three times.

### Measurement of caspase-3 activity

The caspase-3 activity in parental CGTH-W3 and 8505C cells and transfected sublines were measured by using Caspase-3 Activity Assay Kit (Beyotime Biotech, Cat. # C1116) according to the manufacturer’s instructions. The assay is based on the hydrolysis of the peptide substrate acetyl-Asp-Glu-Val-Asp p-nitroanilide (Ac-DEVD-pNA) by caspase-3, resulting in the release of a pNA moiety. Absorbance values were measured at 405 nm. Results were adjusted to the total protein content, and activity was expressed as μmol pNA/h/mg of total protein.

### Scratch-wound migration and transwell invasion assays

Wound healing assays were used to determine cell migration. Briefly, cells grown in 6-well plates as confluent monolayers were mechanically scratched by using a 200 μL pipette tip and then washed with PBS to remove the debris. Cells were cultured for 24 h to allow wound healing. Each scratch-wound area was calculated using the ImageProPlus 6.0 program (Media Cybernetics Inc., Bethesda, MD). Transwell invasion assays were performed with Matrigel (BD Biosciences) coated on the upper surface of the transwell chamber (Corning). Twenty four hours later, cells invaded through the Matrigel membrane were fixed with 4 % paraformaldehyde and stained with crystal violet. The number of invaded cells was counted for analysis.

### RNA extraction and quantitative real-time polymerase chain reaction (qRT-PCR) analysis

Total RNA in cultured cells was isolated using Trizol reagent (Invitrogen, Cat. # 15596–026) following the manufacturer’s instructions, and stored at −80 °C. RevertAid^TM^ First Strand cDNA Synthesis Kit (Fermentas, Cat. # K1622) was used for reverse transcription. qRT-PCR was performed in the ABI PRISM 7500 Sequence Detection System (Applied Biosystems, Foster City, CA) using the SYBR Green RT-PCR kit (Qiagen, Cat. # 204147). All values were normalized using an internal reference (U6, for miRNAs; and GAPDH, for mRNAs). Relative expression was estimated by the comparative Ct method (2^-ΔΔCt^) [18]. A 2^-ΔΔCt^ >3 or < 0.3 was deemed to indicate statistical significance.

### Western blot analysis

Equal amounts of cell lysates were separated by 12 % SDS-PAGE, and electrophoretically transferred to PVDF membrane. The membrane was blocked and probed with primary antibody (anti-ASK1, anti-phospho-ASK1, anti-p38, anti-phospho-p38, anti-ERK1/2, anti-phospho-ERK1/2, anti-ERK5, anti-phospho- ERK5, anti-JNK, anti-phospho-JNK from Cell Signaling Technology; anti-MEKK2, anti-NIS, anti-TSHR from Santa Cruz; anti-β-actin from Sigma) followed by HRP (horseradish peroxidase)-labeled goat anti-mouse IgG (Abcam) or HRP-labeled goat anti-rabbit IgG (Abcam). Chemiluminescence was used to analyze protein levels and β-actin was used as a protein loading control. Semi-quantitative analysis was conducted by using ImageJ 1.49v (National Institutes of Health, USA).

### In vitro iodine uptake assay

Parental CGTH-W3 and 8505C cells and transfected sublines were seeded in 24-well plates at 5 × 10^4^ per well over night. After washed with 1 mL HBSS twice, 1 mL HBSS containing 0.1 μCi Na^125^I and 1 μmol/L NaI was added. After 30 min at 37 °C in a humid atmosphere, cells were collected and washed with ice-cold HBSS. Radioactivity was counted in a γ-counter.

### Statistics analysis

Comparisons of continuous variables between two groups were performed using the Student’s *t* test while categorical variables were performed using the Chi-square test. (a *P* < 0.05 was considered a statistically significant difference). Analyses were performed using the Statistical Package for the Social Sciences, version 20.0 (SPSS, Chicago, IL, USA) and Graph Pad prism Version 5.0 (GraphPad Software, Inc, USA).

## Results

### miR-106a is up-regulated in the serum of patients with non-^131^I-avid lung metastases

**Fig. 1:**
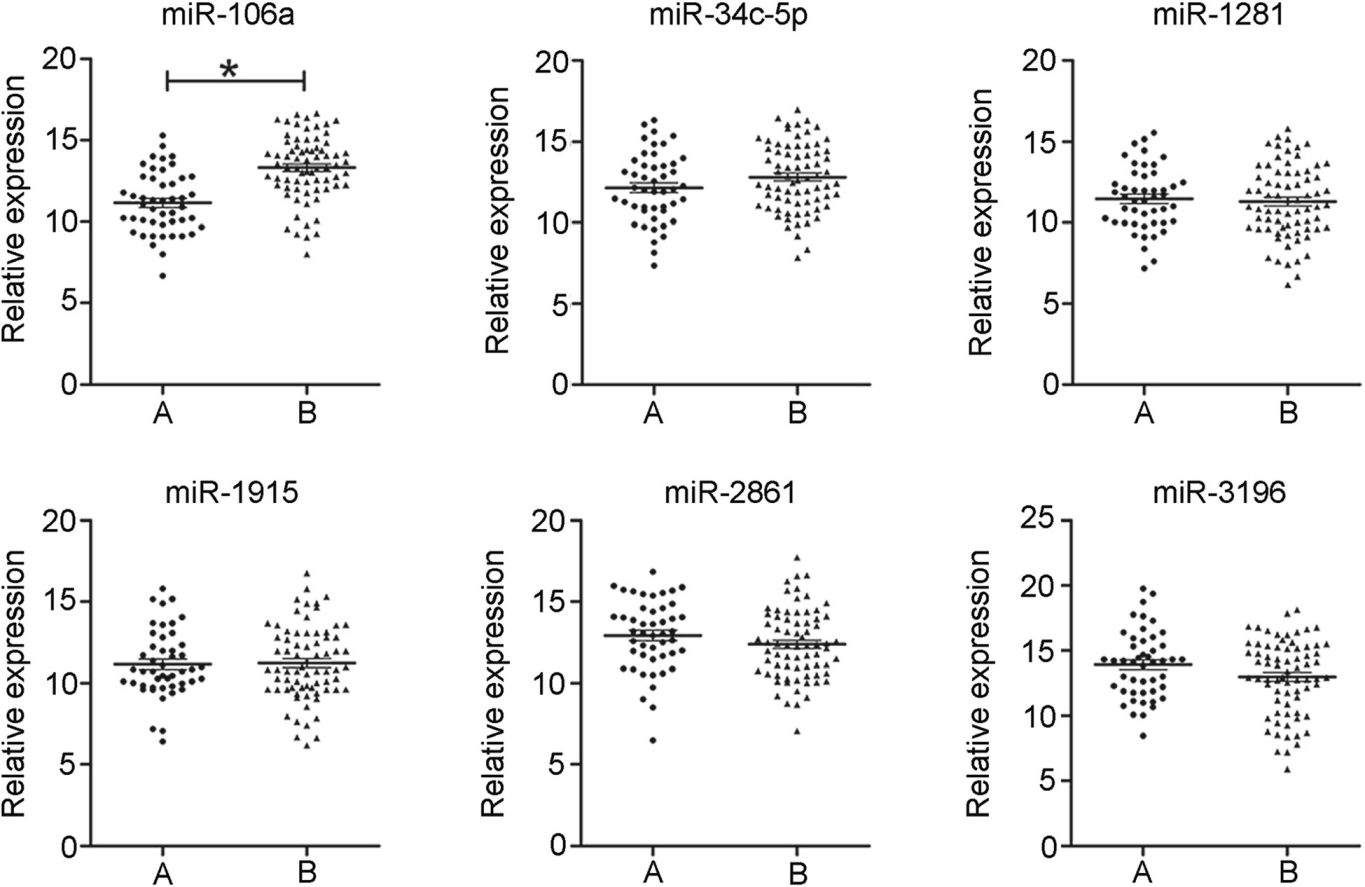
The results of qRT-PCR (ΔCt value) of the six candidate miRNAs (miR-106a, miR-34c-5p, miR-1281, miR-1915, miR-2861, miR-3196). Smaller ΔCt value indicates higher expression. (*, *p* < 0.01)

Forty seven PTC patients with non-^131^I-avid lung metastases (A) and 72 with ^131^I-avid lung metastases (B) were identified in our institution. Demographic and clinical features of these patients are summarized in Table 1. Six candidate miRNAs (miR-106a, miR-34c-5p, miR-1281, miR-1915, miR-2861, miR-3196) which were most changed between the serum of patients with non-^131^I-avid lung metastases or ^131^I-avid lung metastases [12] were validated in the current study. The results of qRT-PCR confirmed the up-regulation of miRNA-106a (*P* < 0.01). However, the expressions of the other five miRNAs had no significant differences between the two categories of patents (Fig. 1).

**Table 1:** Clinical and demographic features of patients with non-^131^I-avid and ^131^I-avid lungs metastases

Clinical Characteristics	Groups		*P* value
	non-^131^I-avid (*N* = 47)	^131^I-avid (*N* = 72)	
Age/years			0.55
Median (range)	43 (28–60)	42 (27–55)	
Gender			0.90
Male (%)	21 (44.7)	33 (45.8)	
Female (%)	26 (55.3)	39 (54.2)	
Tg level (ng/ml)			0.77
Median (range)	1213.5 (304.7–3121.9)	1324.3 (324.9–3249.5)	
TgAb level (IU/ml)			0.72
Median (range)	45.6 (10–125.6)	43.8 (10–150.3)	
Histological diagnosis	PTC	PTC	–
Primary tumor			–
T stage			–
T1	17	27	–
T2	19	30	–
T3	9	12	–
T4	2	3	–
N stage			–
N0	0	0	–
N1	46	70	–
Nx	1	2	–
Chest CT scan	Diffuse small nodules	Diffuse small nodules	–
^131^I up take in lungs	Close to upper limbs	Diffuse lung uptake	–
Extent of disease	No other metastasis	No other metastasis	–
Other cancer history	No	No	–
Other treatment	No	No	–

### miR-106a is differently expressed in different thyroid cancer cell lines and altered after transfection

**Fig. 2:**
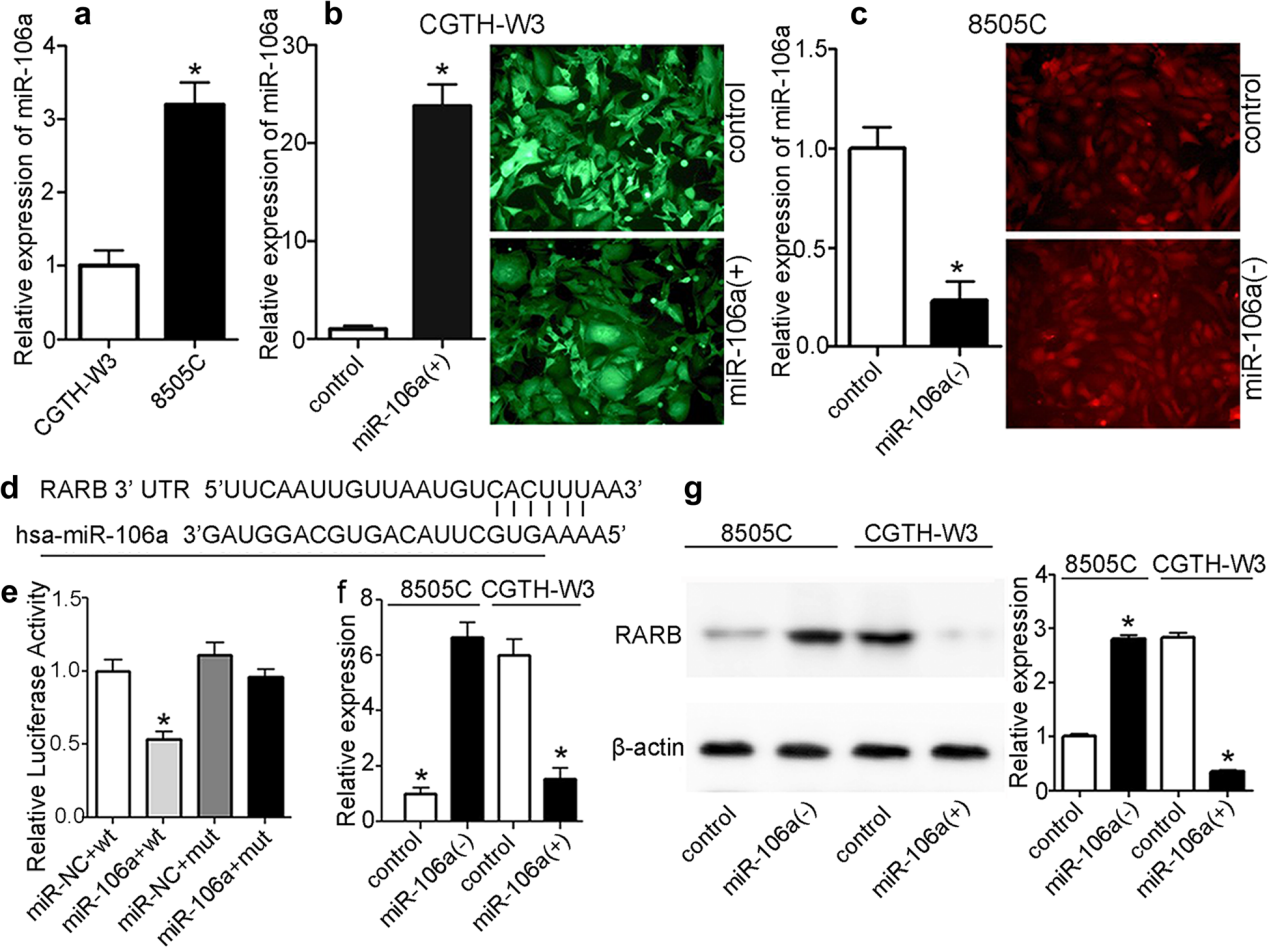
**a**, relative expression of miR-106a in CGTH-W3 and 8505C; **b**, miR-106a overexpression models [CGTH-W3- miR106a(+)]: CGTH-W3 cells transfected with lentiviral vectors carrying the miR-106a gene; **c**, miR-106a knockdown models [8505C-miR106a(−)]: 8505C cells transfected with miR-106a inhibitor gene; **d**, the seed structure of the 3′ UTR of RARB and the miR-106a binding site; **e**, results of dual-luciferase reporter assays; **f**, RARB mRNA expression in different management of cells detected by qRT-PCR; **g**, RARB protein expression in different management of cells by western blotting. (*, *p* < 0.01)

Expression levels of miR-106a were detected in CGTH-W3 and 8505C cells by qRT-PCR. The result showed that the level of miR-106a in 8505C was up-regulated when compared to CGTH-W3 (Fig. 2a). miR-106a overexpression models in CGTH-W3 cells [CGTH-W3- miR106a(+)] and miR-106a knockdown models in 8505C cells [8505C-miR106a(−)] were established by infected with lentiviral vectors carrying the miR-106a gene and miR-106a inhibitor gene, respectively (Fig. 2b-c). The data of qRT-PCR indicated that levels of miR-106a in CGTH-W3- miR106a(+) cells was significantly up-regulated while the level of miR-106a in 8505C-miR106a(−) cells was significantly down-regulated.

### miR-106a directly targets RARB 3′-UTR (miR-106a-RARB)

The 3′-UTR of RARB mRNA contains a complementary site for the seed region of miR-106a (Fig. 2d). To determine whether RARB is a direct target of miR-106a, the RARB 3′-UTR and the mutant containing the miR-106a binding sites were subcloned into a reporter vector downstream of the luciferase gene. Dual-luciferase reporter assays showed that the relative luciferase activity of the reporter that contained wild-type 3′-UTR of RARB mRNA was significantly decreased in miR-106a-overexpressing cells compared with control cells. However, mutation of the predicted binding site of miR-106a on the RARB 3′-UTR rescued the luciferase activity (Fig. 2e). Furthermore, the results of qRT-PCR and western blotting showed that overexpression of miR-106a significantly decreased the expression level of RARB, whereas inhibition of miR-106a induced reduction of RARB mRNA and protein (Fig. 2f, g).

### miR-106a-RARB promote the viability of thyroid cancer in vitro

**Fig. 3:**
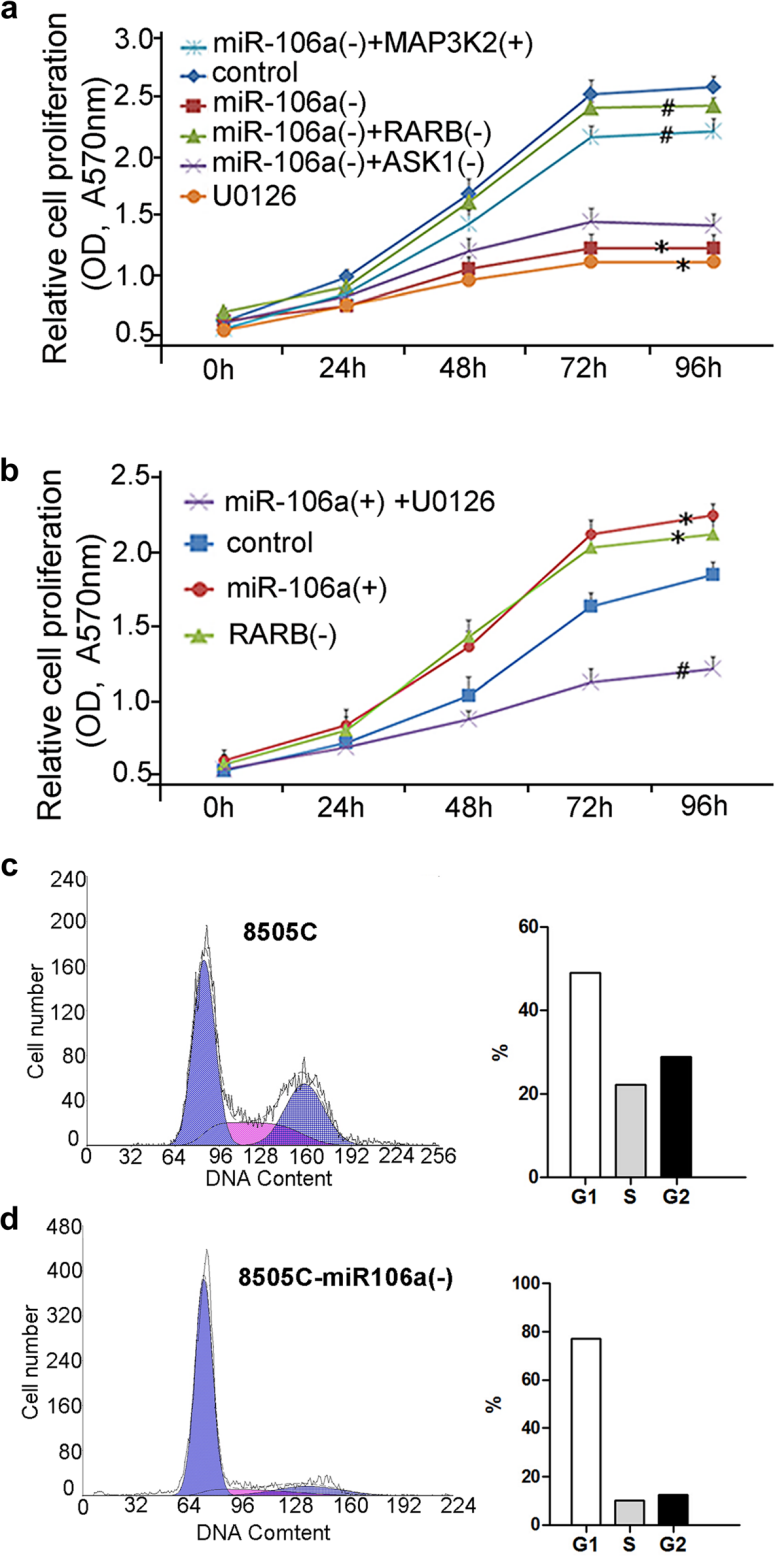
**a**, MTT Cell viability assays: relative cell viability in parental 8505C and transfected sublines [*, *P* < 0.01, miR106a(−) and U0126 vs. control; #, *P* < 0.01, miR106a(−) + RARB(−) and miR106a(−) + MAP3K2 vs. miR106a(−)]; **b**, MTT Cell viability assays: relative cell viability in parental CGTH-W3 and transfected sublines [*, *P* < 0.01, miR106a(+) and RARB(−) vs. control; #, *P* < 0.01, miR106a(+) + U0126 vs. miR106a(+)]; **c** and **d**, flow cytometry analysis of cell cycle in 8505C and 8505C-miR106a(−)

The results of MTT assays demonstrated that overexpression of miR-106a or inhibition of RARB could promote cell viability in CGTH-W3 cells while inhibition of miR-106a could suppress cell viability in 8505C cells (Fig. 3a, b) and the reduced proliferation effect could result from cell cycle arrest (Fig. 3c).

**Fig. 4:**
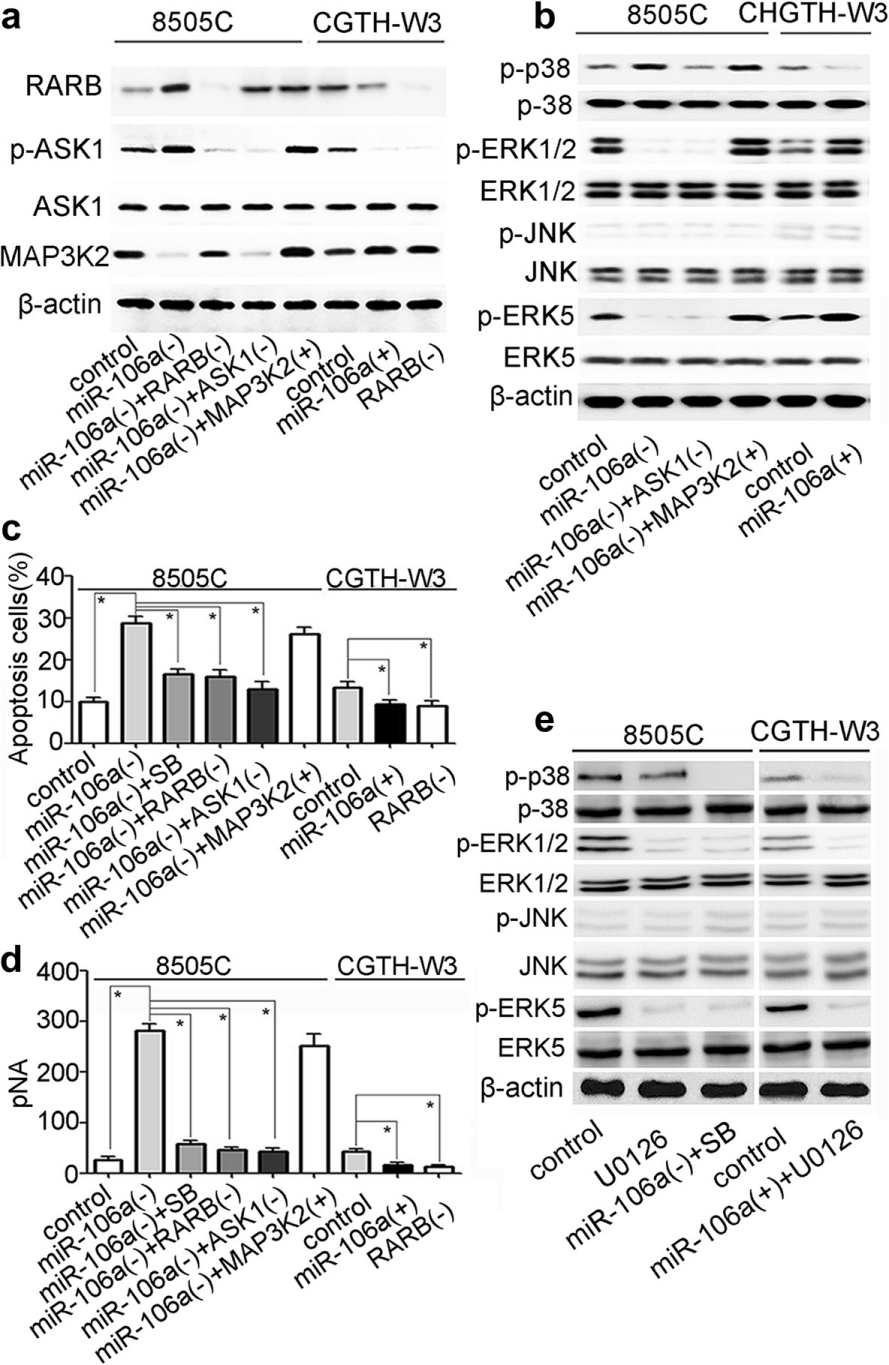
**a**,**b**,**e** western blotting analysis of parental CGTH-W3 and 8505C cells and transfected sublines for proteins in MAPK signaling pathway (SB, SB203580. *, *p* < 0.01); **c**, flow cytometry analysis:apoptosis in parental CGTH-W3 and 8505C cells and transfected sublines (*, *p* < 0.01); **d**, caspase-3 activity in parental CGTH-W3 and 8505C cells and transfected sublines (*, *p* < 0.01)

In order to investigate the potential mechanism of miR-106a-RARB regulating the viability of thyroid cancer cells, 8505C-miR106a(−) cells with overexpression of MAP3K2 (MEKK2) were used. And U0126 (10 μM), an inhibitor of ERK1/2 and ERK5 (Fig. 4e), was used to determine the significance of signaling pathway (as an effect of losing function of ERK1/2 and ERK5). The results of MTT assays showed that 8505C-miR106a(−) cells with overexpression of MAP3K2 promoted cell viability while 8505C and CGTH-W3-miR106a(+) cells treated with U0126 inhibit cell viability (Fig. 3a, b). Western blotting showed that MAP3K2 and its downstream gene ERK1/2/5 were down-regulated in 8505C-miR106a(−) cells when compared to that in 8505C control cells. However, similar to that in 8505C-miR106a(−) + MAP3K2(+) cells, MAP3K2 and its downstream gene ERK1/2/5 were up-regulated in 8505C-miR106a(−) + RARB(−) cells (Fig. 4a, b). In CGTH-W3 cells, MAP3K2 and its downstream gene ERK1/2/5 were up-regulated by overexpression of miRNA-106a or downregulation of RARB (Fig. 4a, b). Together with the results of cell viability assays and western blotting, these data demonstrated that miR-106a-RARB could increase the viability of thyroid cancer cells by activating MEKK2-ERK1/2 and MEKK2-ERK5 pathway.

### miR-106a-RARB inhibit apoptosis of thyroid cancer cells

CGTH-W3- miR106a(+) and 8505C-miR106a(−) cells were used to determine their apoptosis level by flow cytometry analysis. The results of flow cytometry analysis demonstrated that overexpression of miR-106a or inhibition of RARB could reduce apoptosis in CGTH-W3 cells while inhibition of miR-106a could promote apoptosis in 8505C cells (Fig. 4c). And caspase-3 activity of these cells paralleled with the cellular apoptotic vulnerability (Fig. 4d).

Further, in order to investigate the potential mechanism of miR-106a-RARB regulating the apoptosis level of thyroid cancer cells, 8505C-miR106a(−) cells with inhibition of ASK1 by NQDI-1 were used. And SB203580 (20 μM), an inhibitor of p38 (Fig. 4e), was used to determine the significance of signaling pathway (as an effect of losing function of p38). Western blotting showed that p-ASK1 and its downstream gene p-p38 were up-regulated in 8505C-miR106a(−) cells when compared to that in 8505C control cells. However, similar to that in 8505C-miR106a(−) + ASK1(−) cells, p-ASK1 and its downstream gene p-p38 were down-regulated in 8505C-miR106a(−) + RARB(−) cells. In CGTH-W3 cells, p-ASK1 and its downstream gene p-p38 were down-regulated by overexpression of miRNA-106a or down-regulation of RARB (Fig. 4a, b). Together with the results of apoptosis flow cytometry analysis and western blotting, these data demonstrated that miR-106a-RARB could decrease apoptosis of thyroid cancer cells by inhibiting ASK1-p38 pathway.

### miRNA-106a could increase the abilities of invasion and migration in thyroid cancer cells

**Fig. 5:**
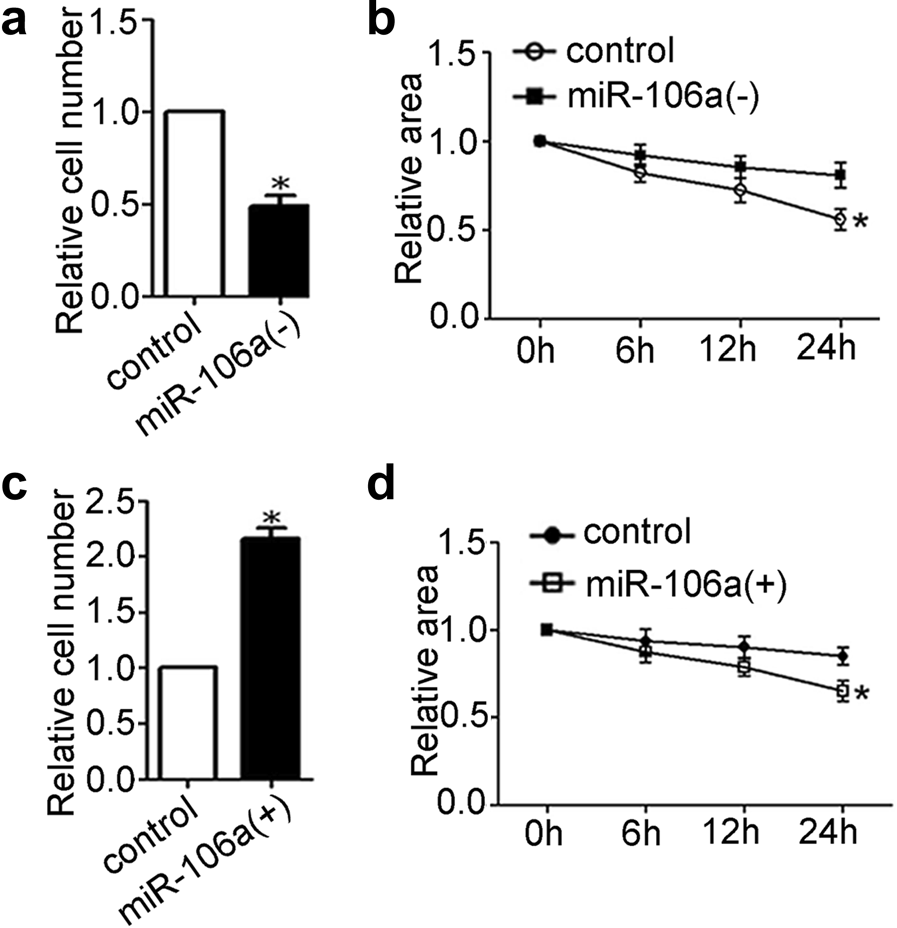
**a**, transwell invasion assay in 8505C-miR106a(−) and control cells (*, *P* < 0.05); **b**, scratch-wound migration assay in 8505C-miR106a(−) and control cells (*, *P* < 0.05); **c**, transwell invasion assay in CGTH-W3-miR106a(+) and control cells (*, *P* < 0.05); **d**, scratch-wound migration assay in CGTH-W3-miR106a(+) and control cells (*, *P* < 0.05)

**Fig. 6:**
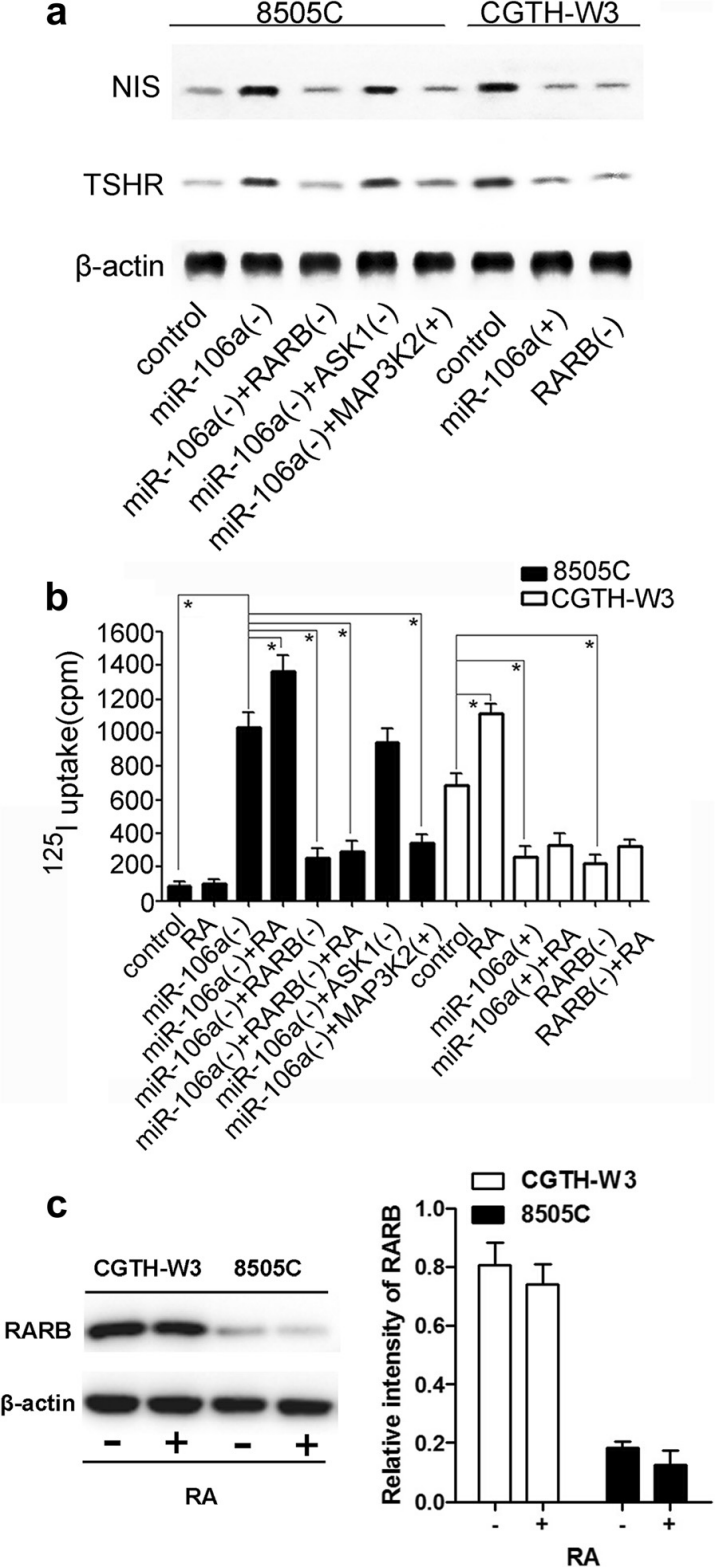
**a**, western blotting analysis of parental CGTH-W3 and 8505C cells and transfected sublines for NIS and TSHR; **b**, iodine uptake ability in parental CGTH-W3 and 8505C cells and transfected sublines (*, *p* < 0.01). **c**, western blotting analysis (together with semi-quantitative analysis of blots) of RARB with and without RA

8505C-miR106a(−) and CGTH-W3-miR106a(+) cells were used to determine the effect of miRNA-106a on the the abilities of migration and invasion in thyroid cancer cells. The results of scratch-wound migration and transwell invasion assays demonstrated that down-regulation of miRNA-106a in 8505C cells could decrease the abilities of invasion (Fig. 5a) and migration (Fig. 5b) while overexpression of miRNA-106a in CGTH-W3 cells could promote the abilities of invasion (Fig. 6c) and migration (Fig. 6d).

### miR-106a-RARB could regulate the expression of NIS, TSHR and alter the iodine uptake function of thyroid cancer in vitro

As showed in the results of western blotting, overexpression of miR-106a or inhibition of RARB could reduce the expression of NIS and TSHR in CGTH-W3 cells, inhibition of miR-106a could increase the expression of NIS and TSHR in 8505C cells and inhibition of RARB or overexpression of MAP3K2 in 8505C-miR106a(−) cells could counteract the effect. However, inhibition of ASK1 in 8505C-miR106a(−) cells did not show the same results [NIS and TSHR expression did not change in 8505C-miR106a(−) and 8505C-miR106a(−) + ASK(−)] (Fig. 6a). Further, the iodine uptake abilities of these cells were studied and the results showed that overexpression of miR-106a or inhibition of RARB could reduce the iodine uptake ability in CGTH-W3 cells. While inhibition of miR-106a could regain the iodine uptake ability in 8505C cells and inhibition of RARB or overexpression of MAP3K2 could counteract the effect (Fig. 6b). In addition, the influence of RA, the ligand of RARB, on ^125^I uptake in these models was also studied and the results demonstrated that RA could increase the ability of iodine uptake in thyroid cancer cells under the existence of RARB (Fig. 6b). Presence or absence of its ligand RA, the expression level of RARB in the cells was not significantly changed (Fig. 6c).

## Discussion

Circulating miRNAs, known as stable cell-free miRNAs in serum or plasma, are passively and selectively released to blood by various cells, and may act as transmitter or messenger in cell communication. During disease, aberrantly expressed miRNAs in the diseased cells are released into the circulation, and the circulating miRNA profile is endued with the disease properties [19]. In the current study, miR-106a detected in patients’ serum could be produced from primary tumors, or metastatic PTC tumor, or even related with tumor micro environment. miR-106a, acting as an Onco-miR, has been found to be associated with carcinogenesis in many carcinomas [20–23] however its role in thyroid cancer has not been reported. miR-106 family members, as the key players in stem cell self-renewal, are highly induced during the early stages of cell reprogramming [24]. miR-106a is known to function in tumor-initiating cells and regulate tumor differentiation through the retinoblastoma (Rb) pathway [22]. Liu et al. reported that miR-106a could specifically repress expression of the retinoblastoma family member RBL2 and miR-106a overexpression resulted in rapid tumor growth and poor differentiation [23]. In the current study, miR-106a, directly targeting RARB, might promote viability of thyroid cancer cells by activating MEKK2-ERK1/2 and MEKK2-ERK5 pathway and increase the apoptosis of thyroid cancer cells by inhibiting ASK1-p38 pathway. Moreover, we also found that miRNA-106a-RARB could regulate the expression of NIS, TSHR and alter the iodine uptake function of thyroid cancer cells through MAPK signaling pathway.

Many signaling pathways have been reported associated with the NIS gene expression and radioiodine uptake in thyroid cancer. Accumulating evidence suggests that aberrant activation of the MAPK pathway plays a central role in the destruction of NIS-mediated iodide accumulation in patients with DTC [25]. The BRAF^V600E^ mutation, one of the key activators of the MAPK pathway, is highly prevalent in recurrent radioiodine-refractory papillary thyroid cancer and is associated with loss of NIS-mediated ^131^I uptake [26–29]. PBF(pituitary tumour-transforming gene [PTTG]-binding factor) is a proto-oncogene that seems to play a crucial part in diminished membrane targeting of NIS [30, 31]. PI3K-AKT-mTOR and NOTCH signaling pathway also have been found to be linked to the regulation of thyroid-specific gene expression. Activation of the PI3K-AKT pathway plays a fundamental role in thyroid tumorigenesis and is involved in downregulation of genes controlling iodide metabolism in patients with DTC [32, 33]. However, overexpression of NOTCH1 in thyroid cancer cells can induce differentiation and stimulate NIS expression [34]. Epigenetic alterations, including DNA hypermethylation and histone deacetylation, also play an important part in silencing thyroid-specific genes, especially NIS [35, 36].

RARB, a member of the thyroid-steroid hormone receptor superfamily of nuclear transcriptional regulators, binds retinoic acid, the biologically active form of vitamin A which mediates cellular signaling in embryonic morphogenesis, cell growth and differentiation. In thyroid cancers, RA induces redifferentiation of cancer cells and expression of the NIS gene. As a result, radioiodine uptake of tumors and serum Tg level are expected to increase with RA treatment [37, 38]. And the loss of retinoid receptors might occur during the loss of differentiation and tumor progression of PTC [39]. Kogai et al. reported that RA stimulation of the NIS in MCF-7 breast cancer cells was meditated by the insulin growth factor-I/phosphatidylinositol 3-kinase and p38 MAPK signaling pathways and an inhibitor of p38 MAPK could significantly reduce iodide uptake in both all-trans retinoic acid-stimulated MCF-7 cells and TSH-stimulated FRTL-5 cells [33].

Some limitations have to be mentioned in the current study. Firstly, the relative expression of miR-106a was statistically different in the serum of PTC patients with iodine avid and non-avid lung metastases, but the difference in the mean levels was modest and there was a large amount of overlap between the groups. In addition, all remaining experiments are in cell models with very large differences in miR-106a expression, not at all modeling the difference found in humans. Secondly, lacking of primary and metastasized tumor tissues (fresh or formalin-fixed and paraffin-embedded sample) made it unable to detect any histotype difference between primary and metastasized tumors and measure miR-106a and RARB in tumor tissues level besides in serum level which could further strength the significance of the findings in the current study.

## Conclusions

In summary, the role of miRNA-106a in the viability, apoptosis, migration, invasion, differentiation and iodine uptake function of thyroid cancer cell lines were investigated in the current research and the results indicated that miRNA-106a directly targeting RARB associated with the viability, apoptosis, differentiation and the iodine uptake function of thyroid cancer cell lines by regulating MAPK signaling pathway in vitro. These findings may provide new strategies for the diagnosis and treatment in radioiodine-refractory DTC.

## Abbreviations

ATC, anaplastic thyroid carcinoma; DTC, differentiated thyroid cancer; MAPK, mitogen-activated protein kinase; NIS, sodium iodide symporter; PTC, papillary thyroid carcinoma; qRT-PCR, quantitative real-time polymerase chain reaction; RAI, radioactive iodine; RARB, retinoic acid receptor beta; TSH, thyroid-stimulating hormone
